# Mechanical and Biological Properties of Magnesium- and Silicon-Substituted Hydroxyapatite Scaffolds

**DOI:** 10.3390/ma14226942

**Published:** 2021-11-17

**Authors:** Sanosh Kunjalukkal Padmanabhan, Paola Nitti, Eleonora Stanca, Alessio Rochira, Luisa Siculella, Maria Grazia Raucci, Marta Madaghiele, Antonio Licciulli, Christian Demitri

**Affiliations:** 1 Department of Engineering for Innovation, University of Salento, 73100 Lecce, Italy; paola.nitti@unisalento.it (P.N.); marta.madaghiele@unisalento.it (M.M.); antonio.licciulli@unisalento.it (A.L.); christian.demitri@unisalento.it (C.D.); 2 Department of Biological and Environmental Sciences and Technologies, University of Salento, 73100 Lecce, Italy; eleonora.stanca@unisalento.it (E.S.); alessio.rochira@unisalento.it (A.R.); luisa.siculella@unisalento.it (L.S.); 3 Institute of Polymers, Composites and Biomaterials-National Research Council (IPCB-CNR), Mostra d’Oltremare pad.20-Viale J.F. Kennedy 54, 80125 Naples, Italy; mariagrazia.raucci@cnr.it

**Keywords:** hydroxyapatite, scaffold, substitution, magnesium, silicon, biocompatibility

## Abstract

Magnesium (Mg)- and silicon (Si)-substituted hydroxyapatite (HA) scaffolds were synthesized using the sponge replica method. The influence of Mg^2+^ and SiO_4_^4−^ ion substitution on the microstructural, mechanical and biological properties of HA scaffolds was evaluated. All synthesized scaffolds exhibited porosity >92%, with interconnected pores and pore sizes ranging between 200 and 800 μm. X-ray diffraction analysis showed that β-TCP was formed in the case of Mg substitution. X-ray fluorescence mapping showed a homogeneous distribution of Mg and Si ions in the respective scaffolds. Compared to the pure HA scaffold, a reduced grain size was observed in the Mg- and Si-substituted scaffolds, which greatly influenced the mechanical properties of the scaffolds. Mechanical tests revealed better performance in HA-Mg (0.44 ± 0.05 MPa), HA-Si (0.64 ± 0.02 MPa) and HA-MgSi (0.53 ± 0.01 MPa) samples compared to pure HA (0.2 ± 0.01 MPa). During biodegradability tests in Tris-HCl, slight weight loss and a substantial reduction in mechanical performances of the scaffolds were observed. Cell proliferation determined by the MTT assay using hBMSC showed that all scaffolds were biocompatible, and the HA-MgSi scaffold seemed the most effective for cell adhesion and proliferation. Furthermore, ALP activity and osteogenic marker expression analysis revealed the ability of HA-Si and HA-MgSi scaffolds to promote osteoblast differentiation.

## 1. Introduction

Treatments for patients suffering from bone defects such as bone ruptures, tumors, abnormalities and osteoporosis very often require the implantation of synthetic biomaterials. Orthopedic reconstructive surgery is exploring a wide range of materials to repair or replace damaged or diseased musculoskeletal tissue, thus aiding in pain recovery and bone regeneration [1,2,3]. An ideal bioactive material should be nontoxic and form bonds with surrounding bone tissue after implantation. Such materials include metals, alloys, polymers and bioceramics [4].

Porous scaffolds for bone regeneration are among the most promising research outcomes of tissue engineering [5]. Ideal scaffolds for bone tissue regeneration require an interconnected and highly porous (>90%) 3D structure to allow cell migration, vascularization and nutrient diffusion [6]. Among the various synthetic scaffolds, bioceramic scaffold composed of hydroxyapatite [HA, Ca_10_(PO_4_)6(OH)_2_], a mineral resembling natural bone and capable of promoting osteoinduction and bone growth, is widely used as maxillofacial, dental and orthopedic bone substitutes [7].

To enhance the bioactivity and biocompatibility of HA, it has been modified with various biologically active cation substitutions [8]. A variety of cationic and anionic substitutions can be attained in apatite minerals. Common substitutional elements are strontium (Sr), magnesium (Mg), silicon (Si), zinc (Zn), copper (Cu), cerium (Ce), fluorine (F) silver (Ag), etc. [9,10]. Kim et al. reported enhanced biocompatibility of magnesium- and silicon-substituted sintered HA and suggested that it could be a useful material for bone augmentation [11]. The processes of bioresorption can be controlled by the substitution of Mg^2+^ and SiO_4_^4−^ ions into the structure of HA, and it also accelerates the formation of a new apatite layer on the surface of the material [12,13,14]. Magnesium also plays an important role in mediating cell–extracellular matrix interaction, thus the incorporation of Mg^2+^ in the HA crystal structure can facilitate new and dense bone formation as a result of the stimulated growth of osteoblasts [15,16]. On the other side, SiO_4_^4−^ ions promote the proliferation and differentiation of cells and the production of collagen by osteoblasts for bone regeneration [17,18].

Owing to the aforementioned properties of Mg^2+^ and SiO^4−^ in bone formation, bioceramics containing Si and Mg have gained attention as a new class of bioceramic materials [19]. Despite the wide interest towards using Mg^2+^ and SiO_4_^4−^ in the field, the synergistic role of these ions towards the bioactivity of porous scaffolds (HA-MgSi) has not been investigated yet. In most works [10,11,16], multi-ion-substituted HA were produced by cosubstituting both ions in the crystal structure of HA, whereas in this work, we substituted Mg^2+^ and SiO_4_^4−^ ions separately during powder synthesis and produced composite scaffolds by mixing the two types of HA powders. The single and combined effects of Mg^2+^ and SiO_4_^4−^ ions for the sintering of highly porous HA scaffolds were investigated in this work. To the best of our knowledge, there are no reports on sintered HA-MgSi composite scaffolds evaluated for physical, mechanical, biodegradation and biocompatible properties.

## 2. Experimental Section

### 2.1. Scaffold Preparation

Pure and substituted (Mg^2+^ and Si^4+^) HA powders with a composition formula Ca_9.6_Mg_0.4_(PO_4_)_6_(OH)_2_ and Ca_10_(PO_4_)_5.3_(SiO_4_)_0.7_(OH)_2_ were prepared by the aqueous precipitation reaction of Ca(NO_3_)_2_·4H_2_O, H_3_PO_4_ (85*w*/*v*), Mg(NO_3_)_2_·6H_2_O, Si(CH_3_CH_2_O)_4_ (TEOS) with a NH_4_OH precipitant, as reported in our previous work [20]. All reagent were purchased from Sigma Aldrich, Milan, Italy. Precipitates were calcined at 900 °C in air. The weight percentage substitution of Mg and Si was 1 and 2 wt %, respectively. Hydroxyapatite scaffolds with Mg^2+^ and Si^4+^ substitutions (HA, HA-Mg, HA-Si and HA-MgSi) were prepared using calcined powders with the sponge replica method [21]. In all slurries, the powder was added up to a final fraction of 70 wt %, and 1 wt % polyvinyl alcohol (PVA, Sigma Aldrich, Milan, Italy) was used as a binder. A polyelectrolyte dispersant (Dolapix CE64, Zschimmer-Schwarz, Lahnstein, Germany) was added as a deflocculating agent, and the mixture was milled in a planetary mill using zirconia balls to obtain a suitable, ceramic suspension for infiltration. The polyurethane (PU) templating sponges (density of 30 kg/m^3^, ORSA Foam S.p.A, Gorla Minore, Italy) were cut into cubes and into cylinders, impregnated with slurries, gently squeezed to remove excess slurry from the pores and dried at 60 °C. The green scaffolds were sintered at 1300 °C for 3 h.

### 2.2. Characterization

A transmission electron microscope (Tecnai G2 Spirit TWIN, FEI Company, Hillsboro, OR, USA) was used to analyze the size and shape of calcined powders.

X-ray diffraction (XRD) analysis was performed on sintered scaffolds using a D-Max/Ultima diffractometer (Rigaku, Tokyo, Japan) and CuKα radiation (λ = 1.5418 Å) in the step scanning mode recorded in the 2θ range of 20–60°. The percentage of secondary phases in synthesized scaffolds was evaluated from XRD data according to the following equation:

(1) $$\nu \;secondary\;phase=\frac{\;\left(I_{1}+I_{2}\right)\;}{\left(I_{HA}+I_{1}+I_{2}\right)}$$

where *I*_1_ and *I*_2_ represent the intensity of the highest peaks present in secondary phases, while *I_HA_* is the intensity of the highest peak of HA. To investigate the homogeneous distribution of Mg and Si ions in the scaffold, mapping was performed using a micro X-ray fluorescence spectrometer (Bruker M4 Tornado, Berlin, Germany).

The linear shrinkage of the scaffolds was evaluated by measuring the dimensions of the sample before and after the sintering process, and apparent density was calculated (weight(g)/volume (mm^3^)).

Porosity Φ of the sintered scaffolds was calculated using the equation:

(2) $$\Phi =1-\frac{\rho }{\rho_{0}}$$

where *ρ* is the apparent density, and *ρ*_0_ the material density of the skeleton material, of which the porous material is made (for hydroxyapatite *ρ*_0_ = 3.16 g/cm^2^). Morphological and microstructural analyses were carried out using a scanning electron microscope (SEM-Zeiss, Berlin, Germany). From the SEM micrographs, the pore size ranges and average grain size of scaffolds were obtained using the ImageJ software.

Mechanical properties of the scaffolds were evaluated by compression tests using a standard testing machine (Lloyd LR5K instrument, Fareham Hants, UK) equipped with a 1 kN load cell [21]. Tests were performed at a crosshead speed of 0.5 mm/min. Six samples for each scaffold’s batch were tested to obtain the stress at failure (σ_max), and the results were expressed as average value ± SD.

### 2.3. Biodegradation Test

To simulate the scaffolds’ behavior in physiological conditions, the samples (six samples for each batch) were immersed in 50 mL of Tris-HCl (Trizma base, 0.05 M’ NaCl, 0.15 M; sodium azide, 0.01% *w*/*v*; pH 7.4) at 37 °C (Julabo GmbH, Seelbach, Germany). The solution was buffered to pH 7.4 with 1 M HCl at selected time points (3, 7, 14 and 28 days). The samples were recovered and gently washed with water and ethanol several times before drying. All samples were dried in an oven at 60 °C for 24 h, and the final weight of each sample was taken [9]. The percentages of weight loss were calculated by:

(3) $$\%\;Weight\;loss=\frac{W_{i}-W_{f}}{W_{i}}$$

where $W_{i}$ is the initial weight of the sample, and $W_{f}\;$ the final weight of the sample after soaking in Tris solution [22]. The results were expressed as average value ± SD.

The mechanical properties of the scaffolds after soaking in Tris buffer at different time intervals were evaluated by uniaxial compression tests. Stress at failure (σ_max) was calculated, and the results were expressed as average value ± SD.

### 2.4. Cell Culture and Proliferation Assay

Human Bone-Marrow-Derived Mesenchymal Stem Cells (BMSC, ATCC-PCS-500-012) and Mesenchymal Stem Cell Growth Kit for Bone-Marrow-Derived MSC containing FGF-b and IGF-1 (ATCC PCS500041) were purchased from ATCC (Milan, Italy).

BMSCs were cultured in low-glucose DMEM supplemented with 10% FBS, 100 IU/mL penicillin/streptomycin, 2.4 mM, 125 pg/mL FGF-b and 15 ng/mL IGF-1 (basal medium) at a density of 5 × 10^3^ cells/cm^2^ and incubated for 24 h at 37 °C under 5% CO_2_. BMSCs were used between the third and sixth passages. Scaffolds were sterilized under UV light overnight, followed by 75% ethanol for 1 h, washed with PBS for 1 h and then incubated with culture medium overnight. Cells were seeded on the top of each scaffold at 2 × 10^4^ cells per scaffold in a final volume of 50 µL. After 1.5 h, the culture medium was added to cover the scaffolds. The medium was changed every 3 days. Cell proliferation was determined using the 3-(4, 5-dimethylthiazolyl-2)-2,5-diphenyltetrazolium bromide (MTT) assay at different time points. MTT is a commonly used method to evaluate the presence of metabolically viable cells, based on the ability of viable cells to convert MTT, a soluble tetrazolium salt, into an insoluble formazan precipitate, which is quantitated spectrophotometrically. Briefly, the scaffolds were transferred into new 24-well plates, and 0.5 mL of culture medium containing 50 μL of MTT solution and 5 mg/mL of phosphate-buffered saline (PBS) solution were then added to each well. After 2 h of incubation, the MTT solution was removed, scaffolds were washed with PBS and 0.5 mL of 0.01 N HCl in isopropyl alcohol was added to solubilize formazan crystals. Absorbance was measured at 570 nm by a spectrophotometer (Beckman Coulter, Brea, CA, USA).

### 2.5. Confocal Microscopy Analysis

For confocal analysis, BMSC were seeded on thin slices of scaffolds (1 mm thickness) at 2 × 10^4^ cells per scaffold in a final volume of 50 µL. After 1.5 h, the culture medium was added to cover the scaffolds. After 7 days, the medium was removed, and the scaffolds with cells were washed twice with PBS and incubated with DiIC18, a fluorescent lipophilic membrane stain. Images of fluorescent-labeled cells were captured by using a confocal laser scanning microscope (CLSM) (Zeiss, LSM 700, Oberkochen, Germany) equipped with a laser diode emitting at 405 nm, an argon-ion laser for excitation at 488 nm and a helium-neon laser for excitation at 514 nm. The DAPI fluorescent signal (in blue) was revealed with a 415–500 nm bandpass filter, and DiIC18 (in red) was evidenced by a 565–660 nm bandpass filter.

### 2.6. ALP Activity Assay

Undifferentiated BMSC were seeded on the scaffolds at a density of 4 × 10^4^ cells/mL and on the well plates at a density of 2 × 10^4^ cells/mL. Cells were cultured in basal medium and incubated for 24 h at 37 °C under 5% CO_2_. The medium was replaced at a rate of 50% every 3 days. BMSC seeded on well plates without scaffolds were used as a control. Alkaline phosphatase (ALP) activity was measured by using the ALP assay kit (MyBioSource, San Diego, CA, USA), according to the manufacturer’s instructions, at 7, 14 and 21 days after seeding. Cell-loaded scaffolds were washed twice with PBS and homogenized mechanically in cold PBS to break cells fully. The enzymatic activity of ALP was monitored through the conversion of 4-aminopyrine in a red quinone derivative. The absorbances were detected at OD = 520 nm. The activity was normalized with respect to the total proteins, and the values of enzyme activity were expressed in unit/mg proteins. ALP activity unit is defined as the amount of 1 mg of phenol produced by 1 mg of proteins reacting with the substrate in 15 min.

### 2.7. Real-Time PCR

Total RNA was extracted from three scaffolds for each condition using TRI reagent ((Sigma, Merck Life Science S.r.l., Milan, Italy), following the manufacturer’s protocol. The reverse-transcriptase reaction (20 μL) was carried out using 1 μg of total RNA, random primers and MultiScribe^®^ Reverse Transcriptase (Applied Biosystem, Monza Italy), according to the manufacturer’s protocol. Quantitative gene expression analysis was performed in a CFX Connect Real-Time System (Bio-Rad) using SYBR Green technology (FluoCycle-Euroclone, Milan, Italy). Primers used in the real-time PCR are reported in Table 1. The efficiency of each primer was tested by running a standard curve in duplicate. The quantifications were performed using the ΔΔCT method, and the *Gapdh* gene was used as an internal control for normalization. Fold change in mRNA expression was relative to the control, represented by BMSC seeded into well plates without a scaffold. The specificity of PCR products was confirmed by melting curve analysis. The identity of the amplified products was confirmed by sequencing analysis.

### 2.8. Statistical Analysis

Values were expressed as mean ± standard deviation (SD) for the indicated number of experiments. Differences between two groups were settled by the unpaired Student’s *t*-test. In all comparisons, *p* < 0.05 was considered statistically significant.

## 3. Results and Discussion

**Figure 1:**
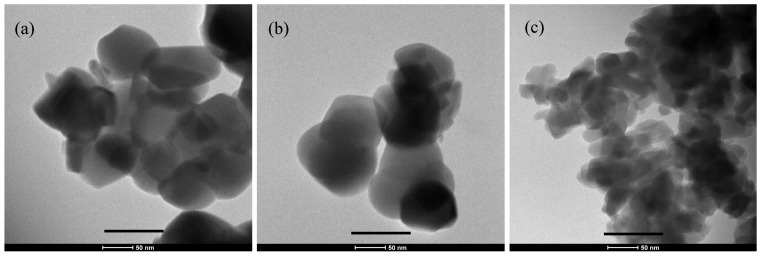
TEM micrograph of synthesized (**a**) HA, (**b**) HA-Mg and (**c**) HA-Si (scale bar = 100 nm).

From the TEM micrographs shown in Figure 1, the particles’ size and shape can be evaluated for the different compositions. Size is influenced by the modification of HA with Mg^2+^ and SiO_4_^4−^ ions. Substitution with Mg^2+^ ions does not change the particle size with respect to pure HA, whereas SiO_4_^4−^ ion substitution resulted in a decrease in the size of the HA particles. This change in size and shape of HA particles with Si substitution has been reported previously [23]. Pure HA- and Mg-substituted powders were prolate spheroidal in shape, 100 nm in size, and for Si-substituted powder, particles changed from prolate spheroidal to elongated in structure with a size of 50 nm.

**Figure 2:**
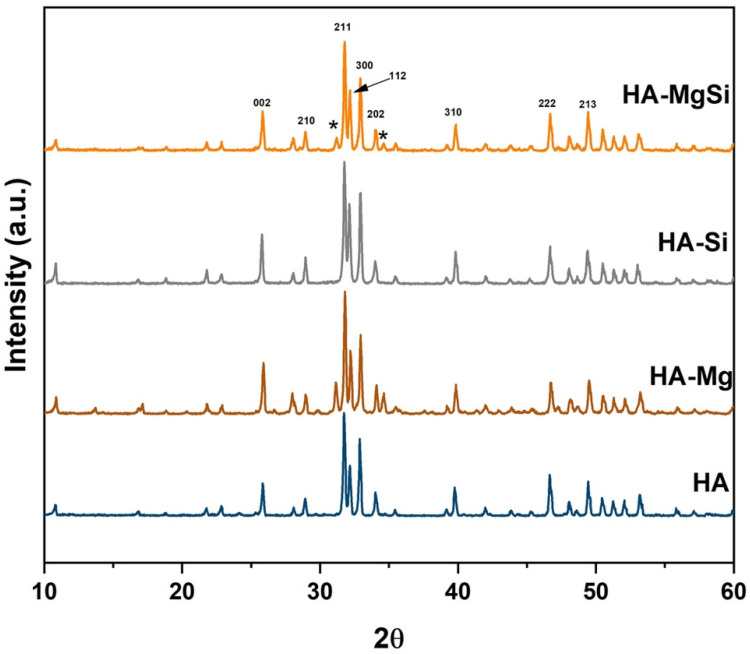
XRD spectra of scaffold sintered at 1300 °C.

The XRD pattern on synthesized powders is reported in our previous work [20]. Briefly, magnesium substitution in the HA crystal structure resulted in β- tricalcium phosphate (β-TCP) formation, whereas HA and HA-Si powder showed only a hydroxyapatite phase. The XRD patterns of scaffolds sintered at 1300 °C are shown in Figure 2, confirming that for HA and HA-Si scaffolds, peaks corresponding to apatite crystalline phases appeared, and for HA-Mg and HA-MgSi samples, peaks of secondary phase β-TCP appeared (marked * representative peaks) together with the HA phase. The peaks of HA and β-TCP match well with JCPDS Nos. 09-0432 and 09-0169, respectively. In both samples, HA was the primary phase, and the amount of β-TCP, calculated using Equation (1), was 30 and 17% for HA-Mg and HA-MgSi samples, respectively. As reported in the literature, some substituent ions for calcium such as magnesium can induce distortion of the crystalline lattice, causing the formation of a secondary phase [24].

**Figure 3:**
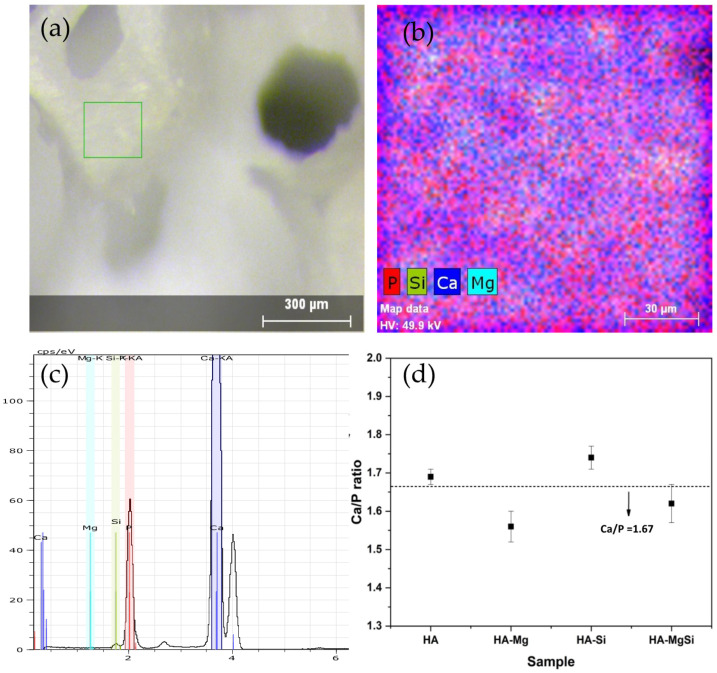
Optical image of mapped zone (**a**), elemental mapping (**b**) XRF spectra of HA-MgSi scaffold (**c**) and plot of Ca/P ratio of all scaffolds obtained from XRF data (**d**).

In order to assess the homogeneity of the distribution of substitutional ions, XRF spectrometry was adopted. In Figure 3a, an optical image of the HA-MgSi scaffold, where the mapping area of the sample with X-ray fluorescence (XRF) spectrometry, is shown. Element mapping and spectra of HA-MgSi scaffolds are shown in Figure 3b,c, respectively. XRF measurements reveal that Mg and Si ions are homogeneously distributed in the respective scaffolds. The calculated weight content of Mg and Si in the respective scaffolds was 0.82% and 1.7%, which is slightly lower than the expected value (1 and 2 wt %, respectively). In Figure 3d, the Ca/P ratio plotted against the representative scaffolds is shown. For HA scaffolds, the Ca/P ratio is almost near to the theoretical value of pure HA (Ca/P = 1.667), whereas for HA-Mg and HA-MgSi scaffolds, the Ca/P ratio decreases due to the formation of β-TCP by the substitution of Mg for Ca. In the HA-Si scaffold, the Ca/P ratio was >1.667 due to the Si substitution for P, and there was no formation of β-TCP.

The linear shrinkage, apparent density, calculated porosity and pore size range of magnesium- and silicon-substituted scaffolds are reported in Table 2. The HA-Mg scaffold shows higher shrinkage behavior compared to all other scaffolds, whereas HA-Si and HA-MgSi scaffolds show almost the same shrinkage with respect to pure HA. All synthesized scaffolds show a low apparent density in the range between 0.21 ± 0.01 and 0.30 ± 0.02 g/cm^3^. All synthesized scaffolds show high porosity (>90%), agreeing with the values reported in the literature, which allows cells to infiltrate, migrate and attach to the scaffold to facilitate bone regeneration. HA-Si showed the highest porosity of 93.1 ± 0.1%.

**Figure 4:**
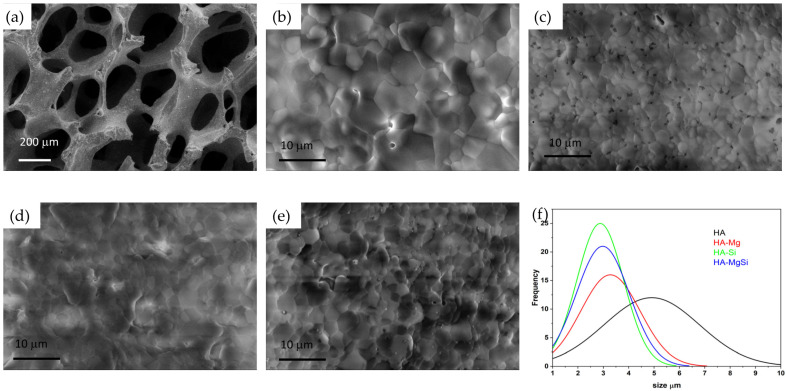
SEM images of the scaffolds sintered at 1300 °C for 3 h. Macrostructure of HA (**a**), microstructure of HA (**b**), HA-Mg(**c**), HA-Si (**d**), HA-MgSi (**e**) and grain size distribution of all scaffolds (**f**).

In Figure 4, the macrostructure of the sintered scaffolds is shown. One can see struts with internal empty cavities deriving from the burn out of the PU sponges used. The sponge struts appeared uniformly impregnated with the ceramic suspension and resulted in a highly interconnected porous structure with a pore size range from 200 to 850 μm. To understand the influence of magnesium and silicon substitutions in HA crystal towards the densification behavior of scaffolds, the microstructure was examined and reported in Figure 4b–e. All scaffolds show a well-defined grain structure with a densified microstructure without any visible defects. The grain size distributions of scaffolds are shown in Figure 3f. Interestingly, we observed that with magnesium and silicon substitution, the average grain size measured showed a decreasing trend compared to pure HA. This may be attributed to the small crystal size of silicon-substituted HA powder (Figure 1c) and grain growth hindering by Mg in the Mg-substituted HA during sintering [25]. The pore size ranges and average grain sizes of scaffolds obtained using the ImageJ software are reported in Table 2.

**Figure 5:**
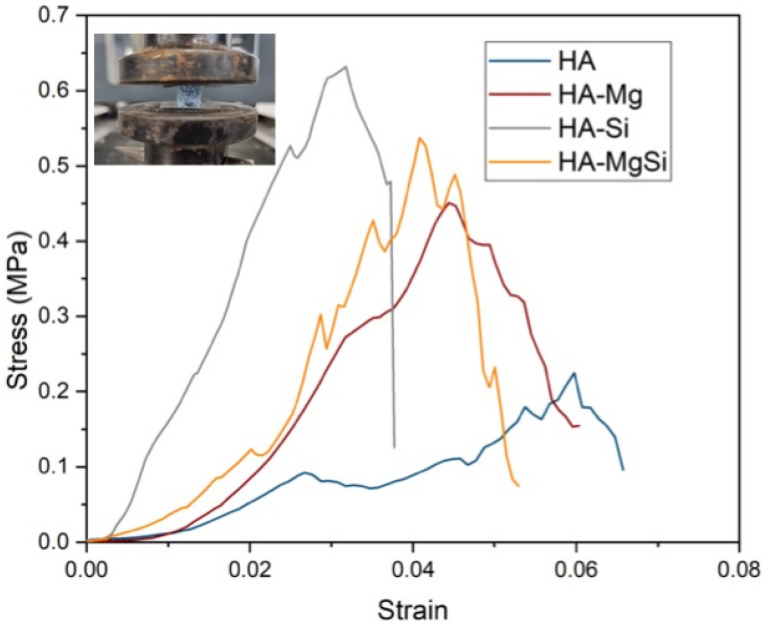
Stress–strain curve of all scaffolds synthesized (inset: sample after compression test).

The mechanical properties of synthesized scaffolds were evaluated by compression tests. Figure 5 represents the stress–strain curves of scaffolds tested. An abrupt increase in stress value is clearly visible at low strain, indicating the fragile nature of scaffolds, which is one of the inherent properties of ceramic materials. Test results revealed that better compression trends are recorded in the case of HA-Mg (0.44 ± 0.05 MPa), HA-Si (0.64 ± 0.02 MPa) and HA-MgSi (0.53 ± 0.01 MPa) compared to HA (0.2 ± 0.01 MPa). All the scaffolds show very small drops in stress during deformation. This small drop is due to the stress failure of struts and not to the failure of the whole structure, and it regains its stress rapidly as the displacement increases. However, these results can also correlate with the microstructural analysis carried out by SEM. In addition to the decrease in grain size, we can observe an increase in the mechanical properties of the scaffolds. The obtained values for mechanical strength are good enough if we relate them to the porosity of the scaffold (>90%) [21].

**Figure 6:**
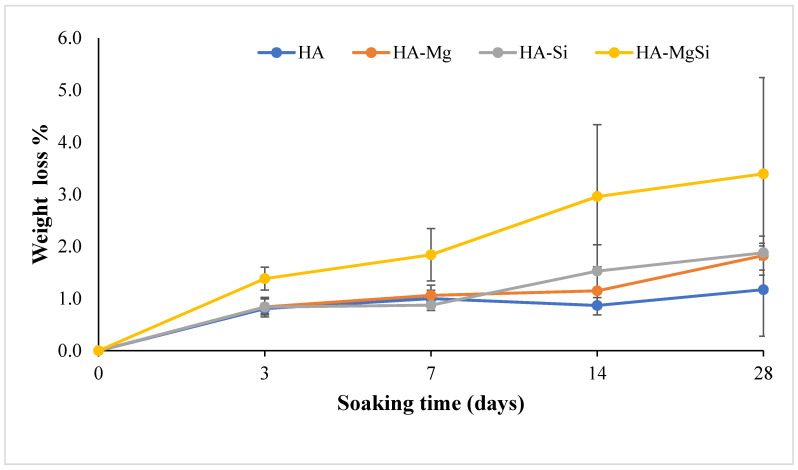
Plot of percentage of weight loss during soaking in Tris-HCl. Average values with standard deviations are reported (n = 6).

To evaluate the biostability, the synthesized scaffolds were immersed in Tris-HCl at 37 °C for different time intervals of 3, 7, 14 and 28 days of immersion. The percentages of scaffold weight change observed during the different time intervals are plotted in Figure 6. It can be noticed that after 28 days, HA-MgSi scaffolds showed higher weight loss around 3.4%, whereas HA showed <1% only. HA-Mg and HA-Si showed almost similar weight loss around 1.8%. These results imply that Mg and Si ion substitution does not alter the biostability of scaffolds much, since the amount of substitution is very less compared to other works [26,27,28].

**Figure 7:**
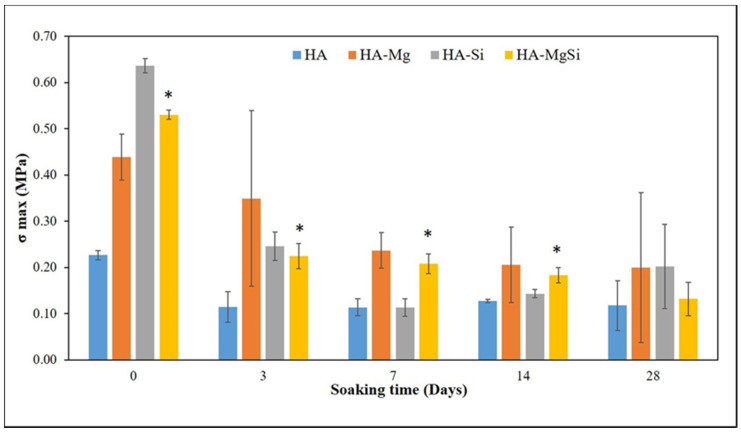
Plot of mechanical strength during soaking in Tris-HCl. Average values with standard deviations are reported (n = 6). * p < 0.05 (HA-MgSi versus HA).

Mechanical strengths of the scaffolds tested after 3, 7, 14 and 28 days of immersion in Tris-HCl solutions are summarized in Figure 7. The trend of compression strength agrees with that found in the scaffold weight variation (Figure 6). The immersion of HA samples in Tris-HCl caused an initial drop in stress at failure and showed constant stress at failure until 28 days of immersion, whereas Mg and Si HA scaffolds displayed a gradual decrease in mechanical properties with increasing time of immersion in Tris-HCl. This scenario can be attributed to the stoichiometric Ca/P molar ratio near 1.67 for pure HA, which is not biodegradable, whereas biodegradability increases with a Ca/P molar ratio higher than 1.67 in the case of impurities or structural defects (HA-Mg, HA-Si and HA-MgSi scaffolds) [22]. At 28 days, the reported weight loss and compression strength showed larger scattering from the mean value, which may be due to the rapid loss of ions from the ceramic strut at a longer incubation time, which varied from sample to sample.

**Figure 8:**
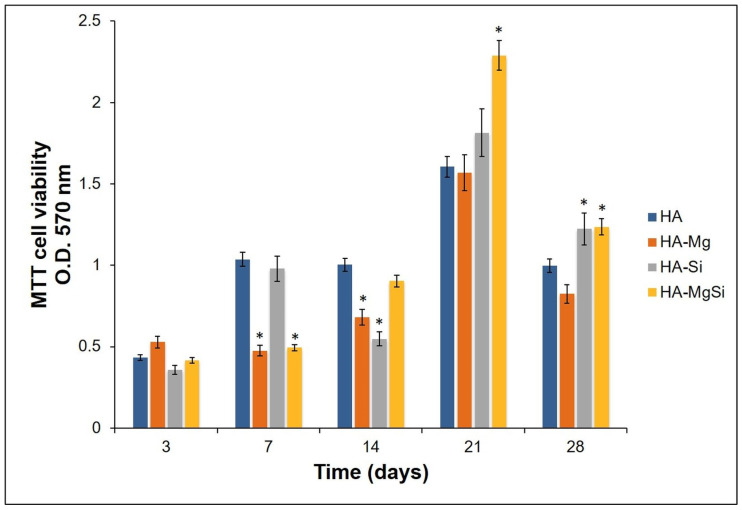
Cell proliferation. BMSC viability was assessed by MTT assay after 3, 7, 14, 21 and 28 days of cell seeding. Data represent the means ± SD of duplicate measurements from three independent experiments. HA scaffold was used as control. * *p* < 0.05.

**Figure 9:**
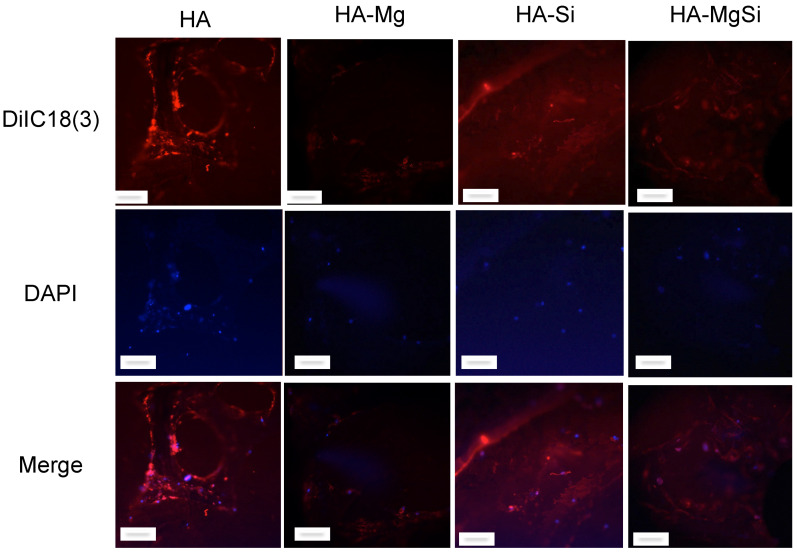
Confocal microscopy images of BMSC seeded on ceramic scaffolds after seven days of culture. Viable cells incorporated DiIC18(3) label (red) into their membrane, and nuclei were stained with DAPI (blue). Individual blue and red channels are shown, followed by merged images (scale bar = 50 μm). Images are representative of three independent experiments.

**Figure 10:**
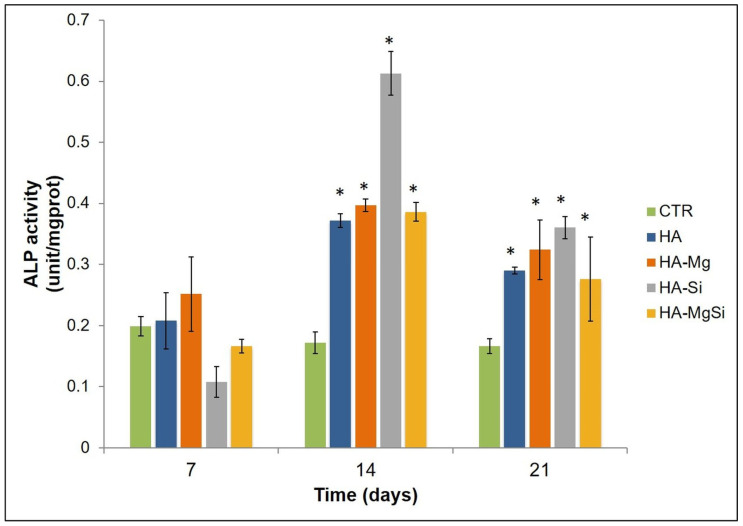
ALP assay. ALP activity was detected in hBMSC seeded on ceramic scaffolds for 7, 14 and 21 days. BMSC seeded into well plates were used as a control (CTR). Results were expressed as the means ± SD of duplicate measurements from three independent experiments (* *p* < 0.01 vs. CTR).

Proliferation of cells seeded on different scaffolds was assessed by measuring metabolic activity through MTT assay. As shown in Figure 8, the numbers of viable and metabolically active BMSC seeded on all the ceramic scaffolds were similar to each other on the third day. The optical density (OD) values increased in HA and HA-Si scaffolds after 7 days. This observation was also confirmed by confocal microscopy analysis (Figure 9). On the scaffold surface, we observed viable cells (in red) that had incorporated DiIC18(3) into the membrane. However, on the 14th day, a slowdown of proliferation was observed in the HA-Si scaffold with respect to the HA scaffold, as already observed in our previous work [20]. The absorbance value of cells seeded in HA, HA-Si and HA-Mg scaffolds on the 21st day increased strongly, more than three times compared to the 3rd day. Moreover, the greatest increment of absorbance was detected in the HA-MgSi scaffold. These results suggested that the HA-MgSi scaffold could be more effective for cell adhesion and proliferation. On the 28th day, we detected a significant reduction of BMSC proliferation into all scaffolds. The decrease in proliferation after 28 days has been investigated. It is known that during cell differentiation the proliferation rate decreased [29,30]. Since several works have shown that HA is able to induce the osteoblastic differentiation of BMSC [31,32], in this study, we investigated the osteogenic potential of different scaffolds. First, we analyzed the effect of different scaffolds on the osteogenic differentiation of BMSC by measuring the activity of ALP. The activity of ALP is important for the cell mineralization process, as this enzyme causes both the local concentration increment of free phosphate and phosphate and calcium active transport across the cell membrane [33]. The enzymatic assay was carried out after 7, 14 and 21 days of cell incubation in basal medium. As shown in Figure 10, all scaffolds determined a significant increase in ALP activity after 14 and 21 days in BMSC incubated in basal medium with respect to control cells, whereas after 7 days, no significant change was detected. However, ALP activity reached maximum values after 14 days of BMSC seeding on the scaffolds, while after 21 days, ALP values showed a decrease while remaining higher than in control cells. These results could depend on the fact that ALP activity reaches the maximum values in the first phase of the osteogenic process, considering that it acts as an early osteogenic marker [34,35]. To go into detail, although all scaffolds seem to determine a significant increment in ALP activity in BMSC cells, the highest activity value was observed in the HA-Si scaffold after 14 days of BMSC seeding.

**Figure 11:**
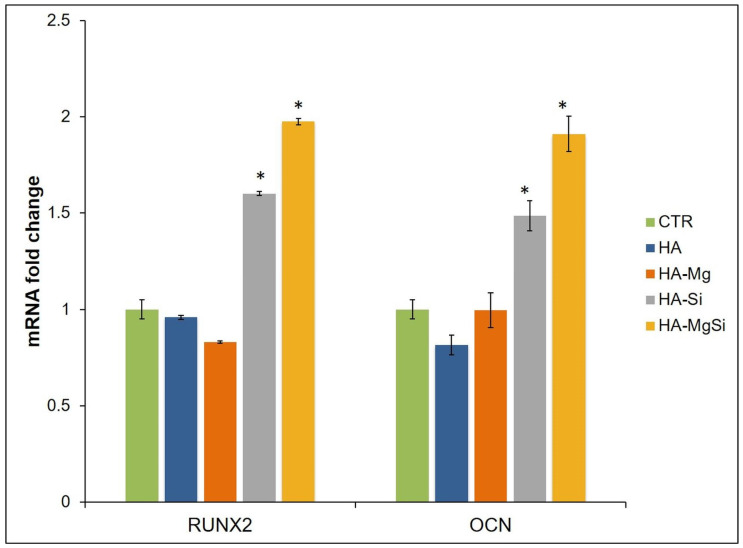
Effect of ceramic scaffolds on osteogenic gene expression. mRNA abundance of RUNX2, OCN in BMSC seeded on scaffolds and cultured in basal medium for 28 days. Gapdh was used as a housekeeping gene for normalization. Fold change in mRNA expression was relative to control (CTR) represented by BMSC seeded into well plates. Results were expressed as the means ± SD of triplicate measurements from three independent experiments (* *p* < 0.05 vs. CTR).

To further investigate the effects of ceramic scaffolds on BMSC osteogenic differentiation, we quantified, by real-time PCR, the mRNA abundance of RUNX2 and OCN, which represented the transcription factor key regulator of osteogenesis and the extracellular matrix protein used as an osteogenic differentiation marker, respectively (Figure 11) [36,37]. HA-Si and HA-MgSi scaffolds induced in BMSC cultured in basal medium for 28 days, a marked increase in the mRNA level of RUNX2 and OCN with respect to CTR. Indeed, RUNX2 expression augmented by about 60% and 49%, whereas OCN mRNA abundance increased by about 98% and 91% in HA-Si and HA-MgSi scaffolds, respectively, when compared to CTR. No significant difference between HA, HA-Mg scaffolds and control was observed in the RUNX2 and OCN mRNA expression.

The increase in ALP activity on the 14th day and of osteogenic marker mRNA levels on the 28th day confirms the ability of HA-Si and HA-MgSi scaffolds to promote osteoblast differentiation. Indeed, since it is known that ALP is involved in extracellular matrix mineralization, its activity rises in the early phase of the osteoblast differentiation process, and then it declines when osteoblasts differentiate to osteocytes. By contrast, RUNX2 and OCN expression increases later, and it is essentially typical of osteoblasts in the post-proliferative phase [36,38]. It has been reported that negatively charged surfaces show better cell attachment, proliferation and osteoblast differentiation than untreated hydrophobic surfaces [39]. These results suggest that the microstructure, mechanical properties and charge features of HA-Si and HA-MgSi play crucial roles in cell adhesion, growth and differentiation, although the phenomenon of cell–surface interaction is very complex; therefore, it is not yet clear which property could be dominant for cell adhesion and growth on surfaces. Taken together, our data demonstrated that these materials not only exhibit excellent performance in terms of biocompatibility and cell viability, but they also could provide a strong osteogenic commitment to mesenchymal stem cells.

## 4. Conclusions

Mg- and Si-substituted HA scaffolds were fabricated from hydroxide coprecipitation combined with the sponge replica method. The single and combined effects of Mg and Si modification on the sintering behavior, microstructure, mechanical strength, biodegradation and biocompatibility were carefully investigated. Substitution of a smaller Mg^2+^ for a larger Ca^2+^ ion induced structural changes and partial decomposition of the HA structure during the heat treatment process with secondary β-TCP segregation. XRF analysis confirmed the homogeneous distribution of Mg and Si ions on each scaffold. All types of scaffolds showed porosity >90% and a pore size ranging between 200 and 850 μm. With magnesium and silicon substitution, the size of crystalline grains is smaller than pure HA, which provides better mechanical properties for Mg- and Si-containing HA scaffolds. Mg and Si ions did not alter the biostability of scaffolds in terms of weight loss, since the cation substitution was very little. MgSi scaffolds exhibit higher mechanical strength; nevertheless, the decrease in strength is more prominent after 28 days of incubation in Tris-HCl, suggesting a faster dissolution mechanism. Cell proliferation experiment using BMSC showed that all scaffolds were biocompatible, and composite scaffold (HA-MgSi) is more effective for cell adhesion and proliferation compared to all other scaffolds. Furthermore, due to their strong osteogenic potential on mesenchymal stem cells, HA-Si and HA-MgSi scaffolds could represent very interesting materials in the field of bone reconstructive surgery.

## Figures and Tables

**Table 1:** Oligonucleotides used for real-time PCR analysis.

Gene Name	Accession Number	Sequences	pb
*RunX2*	NM_001278478.2	F: Gacaaccgcaccatggtgg R: Tctggtacctctccgaggg	160
*Ocn*	NM_199173.6	F: Gctacctgtatcaatggct R: Cgatgtggtcagccaactc	111
*Gapdh*	AJ005371.1	F: Atggccttccgtgtccccac R: Acgcctgcttcaccaccttc	245

*RunX2*, runt-related transcription factor 2; *Ocn,* osteocalcin; *Gapdh,* glyceraldehyde-3-phosphate dehydrogenase.

**Table 2:** Linear shrinkage, apparent density, porosity, pore size ranges and grain size of synthesized scaffolds.

Sample	Linear Shrinkage (%)	Apparent Density (g/cm^3^)	Porosity (%)	Pore Size (μm)	Grain Size (μm)
HA	15	0.23 ± 0.03	92.4 ± 0.1	298–829	4.95 ± 1.5
HA-Mg	18	0.26 ± 0.02	91.7 ± 0.6	203–701	3.28 ± 1
HA-Si	16	0.21 ± 0.01	93.1 ± 0.1	324–847	2.88 ± 0.8
HA-MgSi	14.5	0.30 ± 0.02	90.2 ± 0.2	300–829	2.98 ± 1

## Data Availability

Data is contained within the article.
